# A Crusade against Indifference

**Published:** 1887-06-11

**Authors:** 


### A Crusade against Indifference.

When Demosthenes, the great Athenian orator, was asked what, in his opinion, was the first requisite in speaking, he replied, "Action." Questioned as to the second and third essentials in the orator's art, he still replied "Action." Similarly we would say of public charity that the first, the second, and the third essential is organisation. The magnitude which the hospital system in this country has attained in itself demands that the flow of public alms to the coffers of the hospitals shall be so regulated and guided as to produce the best results. But when we remember that this flow of public alms falls terribly short of the requirements, it is obvious that the need of some system of organisation which shall encourage men to give to one of the grandest causes upon earth—the alleviation of the sickness and the suffering of our poor and helpless fellow-creatures—is greatly accentuated. The givers—those who actually bear the burden of the cost of our hospitals—are few; their numbers are out of all proportion to the population of the country. If there are any who doubt this assertion, they will, we think, be soon convinced if they will take the trouble to analyse the names in the subscription lists of some of our hospitals. This is not as it should be, and hence it is incumbent on every well-wisher of our medical charities to use his personal influence among his friends to enlarge the number of subscribers.

Last year, at the suggestion of the late Dr. Wakley, a series of meetings, extending over a week, was held in different parts of the metropolis, with a view to arousing public attention to the needs of our hospitals, prior to Hospital Sunday. The Hospitals Week was a success, the aggregate of the collections in the churches far exceeding the sum raised in any previous year. The Council of the Hospital Sunday Fund this year have determined, on an extended scale, to again draw the attention of the public to the pressing pecuniary needs of our hospitals, and the importance to all classes of the community, from the highest to the lowest, of their efficient maintenance. We published last week a tabulated list of meetings, extending over the fortnight preceding Hospital Sunday this year, which the Council has arranged for this purpose.

### THE MEETING AT LAMBETH PALACE.

The first of these meetings was held, by kind permission of the Archbishop of Canterbury, in the historic library of Lambeth Palace, his Grace lending his great influence to the movement by occupying the chair. Among those present were Viscount Lewisham, M.P., Lieut.-General Crawford Fraser, V.C., M.P., the Rev. Newman Hall, the Rev. A. W. Jephson, the Rev. Dr. Linklater, D.D., Mr. Henry C. Burdett, of The Hospitals Association, the Rev. J. F. Macdonald, the Rev. Dr. Lee, and Mr. R. Garrard Kestin, the Hon. Sec. Unfortunately the meeting fell on the day of the opening of Parliament after the Whitsuntide recess, which prevented the attendance of some influential speakers. Among others who wrote regretting their inability to attend were the Rev. and Hon. Canon Pelham, the Rev. Erskine Clarke, the Rev. Canon Fleming, Alderman Sir William McArthur, K.C.M.G., Mr. W. Gent-Davis, M.P., General Lowrie, and Mr. Walter Kimber. Sir Edmund Hay Currie, in a letter addressed to the Archbishop of Canterbury, stating that he was prevented from attending, wrote: "It is of vital importance that a good start should be made this day under your guidance. I do not think there ever was a time when a more comprehensive effort should be made than the present year for collections for the maintenance of existing beds, and to enable many more to be opened."

General Fraser, who presided in the temporary absence of the Archbishop, briefly explained the objects of the meeting, and exhorted his hearers to work individually, and to use their influence with all classes for the purpose they had in view. The difficulties which we knew to be ahead of us could be met by energy and enthusiasm in the cause. Enthusiasm was catching, and thus the lesson of doing good might be taught. By inducing people to believe that by being kind and charitable and unselfish and thoughtful for others, they would for themselves be making their own path in life one strewn with flowers and cheered by sunshine. If we raised their spirits to such thoughts, we should enable them to realise that goodness was no name and happiness no mere term.

Viscount Lewisham proposed the first resolution: "That this meeting pledges itself to use every endeavour to arouse the inhabitants of this district of London to the importance of maintaining the hospitals and medical charities in the utmost efficiency. In furtherance of this object it urges the clergy and the ministers of religion to point out the advantage of giving through the Hospitals Sunday Fund, owing to the nominal cost of so collecting funds for hospitals, and to make an earnest appeal to the people to so increase their contributions as to secure that the whole sum collected shall not be less than £100,000." His lordship observed that the hospital movement, great as it had been, had not answered all the anticipations made of it, and it was in this Jubilee year of her Majesty's reign that they hoped to take a new departure to ensure that this movement should attain the ends it was wished to meet. It was calculated that if one relation of every inmate of a hospital, who had derived personal advantage from hospital treatment, were to give a shilling, something like £50,000 would be at once raised. They had had an instance this year of what might be done in the way of raising subscriptions, and he thought that, as this was her Majesty's fiftieth year, they should pledge themselves to this resolution to promote a movement which all had at heart. Lord Lewisham, in conclusion, said that he had been personally connected with hospitals for many years, and he fully realised the importance of having a thoroughly well-worked hospital system.

The Rev. A. Jephson, in seconding the resolution, said that the late Lord Shaftesbury had remarked that the charitable public—the giving public—was a distinctly limited body, and that any person who added to that number was a distinct benefactor to the country in which he lived. The extraordinary appeals which had been made to the public this year, for various purposes in celebration of the Queen's Jubilee, had tended to divert money from the hospitals. Thus many persons who had given to the Church House would otherwise have given to the hospitals, and the Imperial Institute was doing the same kind of thing. All who had hearts and human sympathy would go forward to increase the number of the charitably disposed, and he hoped that if any of his hearers did not subscribe they would begin to do so, or if they could not do so, that they would get some one else to do so.

The Rev. Dr. Lee, in the course of some lengthy remarks, was proceeding to bring charges of extravagance against the General Lying-in Hospital, when the Rev. A. Jephson rose to a point of order. The Archbishop, who had arrived in the meantime, ruled that Dr. Lee was out of order, the meeting having been called for a specific purpose, and not to criticise the management of any particular institution. Dr. Lee sought permission to move an amendment, but the Rev. Chairman declined to allow an amendment of the kind indicated. Dr. Lee said that as his Grace refused to give him a hearing he must obtain it through the newspapers.

The Rev. HERMAN GOLLANCZ, speaking in support of the resolution, said that not only was this noble work of charitable relief, as far as regarded the hospitals, not limited to any district, but it was not limited to any creed or race. As the representative of the Jewish community of that district, Mr. Gollancz remarked that he had for fifteen years cheerfully supported the Hospital Sunday movement. There was an opinion that the Jews were a wealthy body, but, though doubtless there were some very rich ones, there was a great mass of the very poor. His people had a great number of institutions for the relief of their poor, by which they were, as far as possible, prevented from burdening the local charities. But, nevertheless, the Jewish community readily joined with Christians in subscribing to so noble an institution as the Hospital Sunday Fund. The resolution having been unanimously carried,

Mr. H. C. BURDETT moved: "That this meeting regrets to learn that the expenditure and liabilities of the Hospitals and Medical Charities in this district in the year 1886 exceeded the income by more than £3,000, and pledges itself to increased exertion, with the view of preventing any like deficiency during the current year." Before speaking more immediately on the subject-matter of the resolution, Mr. Burdett referred to the object of the institution, of the addition of the Hospitals Fortnight to the Fund. It was, he observed, not a question of benefiting a hospital in any particular locality, but to awaken generally the area of interest. It was with the knowledge that at the present time the London hospitals and other medical charities were in sore distress for funds that this new departure had been taken. The London hospitals as a whole required £40,000 a year more than they received. If it were not for the Hospital Sunday and Saturday Funds there would be a deficiency of £100,000 every year between the expenditure and the receipts.

The Rev. NEWMAN HALL thought that the appeal of the hospitals to the churches was a grand tribute paid to Christianity. It was said that we spent so much money upon building churches, and sending our religion to the uttermost ends of the earth; why did we not do more for our hospitals? But the fact was that the very people who subscribed to the churches were those who subscribed to the hospitals. If the people put a little check upon their extravagance and applied the money thus saved to the hospitals, there would be no necessity for such meetings as these. After alluding to the threatened dangers from socialism, arising from the existence of vast wealth side by side with squalid poverty, the rev. speaker in eloquent terms impressed upon his hearers the importance of bringing all classes to feel that whatever we possessed was not our own to do just as we liked with, but for our wise use. We wanted the poor to be associated with us in the grand enterprise of helping the hospitals; we appealed for the poor man's penny, so that the poorest man in London might feel that he was taking his part in this work with the Archbishop of Canterbury himself. Then, in matters of legislation there should be a large hearted generosity, an earnest interest in the poor to promote their welfare, to diminish their temptations, doing something to give them better habitations, making it difficult for them to go to the bad, and easier to climb to the good. We should take an interest in their social recreations, seeking every possible opportunity to make them feel that, whatever differences there were, there was an equality of sympathy, a brotherhood between the rich and the poor. By these means we should best keep back the inundation of socialism, promote the interests of our hospitals, and celebrate the Jubilee of our Queen, making it a Jubilee of her people too.

The ARCHBISHOP OF CANTERBURY, whose remarks we report elsewhere, next addressed the meeting.

The ARCHBISHOP, in replying to a vote of thanks, proposed by Mr. JOHN HUTTON, Churchwarden of St. John's, Lambeth, and seconded by Mr. JOHN HERNAMAN, paid a well-deserved compliment to Mr. Kestin, the able Secretary to the Hospital for Women and Children, Waterloo Bridge Road, for his services in organising this excellent meeting.

### MEETING AT PADDINGTON.

On Tuesday evening the second meeting was held at the Vestry Hall, Paddington Green, under the presidency of the Vicar, the Rev. Walter Abbott, M.A., who was supported by the Baron Ferdinand de Rothschild, the Hon. Henry Noel, J.P., Mr. H. C. Burdett, Mr. E. Parker Young, Mr. P. Michelli, Dr. Danford Thomas, and the Rev. E. S. Dewick. Among the absentees were Mr. Cohen, M.P., Sir John Kennaway, M.P., and Mr. E. Boulnois, J.P.

The CHAIRMAN, in opening the proceedings, remarked that the cause was one which interested all, both laymen and clergy. He thought that the more the clergy took an interest in the bodies of the people, the better. It was surprising, taking a parish of from six to ten thousand people, to find how few there were who were really ill—who could not go to their work. In such a parish as his, there would perhaps not be a dozen. It was to a large extent a world of health. Many persons went through life with only three or four sicknesses in fourscore years. The hospitals, it appeared, were in difficulties; and, as far as he knew, there were only two ways of getting out of debt—by decreasing expenditure, or by increasing income. We had no reason to believe that the hospitals were extravagant. Here was the importance of the Hospital Sunday Fund, which saw that the hospitals participating were carried on on economical principles. Some people might think it was a very stingy world; possibly it was, but he had seen the other side of it. There was a great deal of generosity, but it was confined to a few people who were found to help everything. The problem was, therefore, how to reach the non-givers. He did not think they could be reached until we put better principles into them. Until they learnt the blessedness of almsgiving, the hospitals must go a-begging, and be in debt.

The BARON FERDINAND DE ROTHSCHILD moved the first resolution in an able speech, which we propose to publish in full.

The Hon. H. NOEL, in seconding the resolution, remarked that the proposer had made a feeling appeal to the Press as to their great power, but he thought the appeal should rather be to the clergy, who might plead from the pulpit the cause of the hospitals, not once a year, but, if they chose, fifty times. He lived in Westbourne Terrace, where there were 160 houses rated at about £250 a year, and therefore inhabited by people who were more or less well-to-do. Out of that number he found only sixty subscribed to the local hospital, so that he felt the clergy had not taken so full an advantage of their position as they might.

Mr. H. C. BURDETT moved the second resolution: "That this meeting regrets to learn that the expenditure and liabilities of the hospitals and medical charities of this district, in the year 1886, exceeded the income by £18,000; and that it pledges itself to increased exertion with the view of preventing any deficiency during the current year."

The Vicar of Paddington expressed the best thanks of the meeting to Mr. P. Michelli, Secretary to St. Mary's Hospital, and to Mr. Thomas Ryan, Secretary to Queen Charlotte's Lying-in Hospital, for the excellent manner in which they had organised the present meeting.

